# Docking Proteins Upregulate IL-1β Expression in Lower Esophageal Sphincter Muscle in Esophageal Achalasia

**DOI:** 10.3390/jcm13103004

**Published:** 2024-05-20

**Authors:** Tsutomu Kanda, Karen Saiki, Hiroki Kurumi, Akira Yoshida, Yuichiro Ikebuchi, Takuki Sakaguchi, Shigetoshi Urabe, Hitomi Minami, Naoyuki Yamaguchi, Kazuhiko Nakao, Haruhiro Inoue, Hajime Isomoto

**Affiliations:** 1 Division of Gastroenterology and Nephrology, Faculty of Medicine, Tottori University, Yonago 683-8504, Japan; 2 Division of Immunology, Faculty of Medicine, Tottori University, Yonago 683-8504, Japan; 3 Digestive Center, Showa University Koto-Toyosu Hospital, Tokyo 135-8577, Japan; 4 Department of Gastroenterology and Hepatology, Nagasaki University Hospital, Nagasaki 852-8501, Japan

**Keywords:** esophageal achalasia, peroral endoscopic myotomy, herpes simplex virus type 1, docking proteins 1 and 2, IL-1β

## Abstract

**Background/Objectives:** Esophageal achalasia is an archetypal esophageal motility disorder characterized by abnormal peristalsis of the esophageal body and impaired lower esophageal sphincter (LES) relaxation. **Methods:** In this study, the mRNA expression of docking proteins 1 and 2 (*DOK1* and *DOK2*, respectively) were analyzed and the mechanisms underlying achalasia onset were investigated. **Results:** *DOK1* and *DOK2* mRNA levels significantly increased in the LES of patients with achalasia. Moreover, significant correlations were observed between *IL-1β* and *DOK1*, *IL-1β* and *DOK2*, *ATG16L1* and *DOK1*, and HSV1-miR-H1-3p and *DOK2* expression levels. However, a correlation between *ATG16L1* and *DOK2* or between HSV-miR-H1-3p and *DOK1* expression was not observed. In addition, a positive correlation was observed between patient age and *DOK1* expression. Microarray analysis revealed a significant decrease in the expression of hsa-miR-377-3p and miR-376a-3p in the LES muscle of patients with achalasia. **Conclusions:** These miRNAs possessed sequences targeting DOK. The upregulation of *DOK1* and *DOK2* expression induces *IL-1β* expression in the LES of achalasia patients, which may contribute to the development of esophageal motility disorder.

## 1. Introduction

Esophageal achalasia is an archetypal esophageal motility disorder characterized by the abnormal peristalsis of the esophageal body and impaired relaxation of the lower esophageal sphincter (LES). Therefore, esophageal achalasia is known to impair eating ability and reduce quality of life [1,2,3].

Currently, the proposed causal factors of esophageal achalasia are diverse and multifactorial and involve complex interactions between autoimmune and inflammatory responses, possibly triggered by viral infections in genetically susceptible individuals [2]. Causal viral agents include the herpes simplex virus—a neurotropic virus with a predilection for the squamous epithelium—varicella zoster virus, measles virus, and human papillomavirus [4,5,6]. Moreover, studies have reported the presence of herpes simplex virus type-1 (HSV-1) DNA and RNA in all tissues obtained from patients with achalasia, whereas these viral components were not detected in control tissues [7]. Therefore, research on HSV-1 infection is crucial for understanding the etiology of achalasia.

MiRNAs are single-stranded RNAs that regulate gene expression and play crucial roles in physiological and pathological processes, including viral infection and hostile antiviral responses [8,9,10,11]. Some viruses, especially herpes viruses such as HSV-1, express miRNAs, although their pathological roles are not entirely understood [8,11].

Our previous study findings revealed significantly higher HSV1-miR-H1-3p expression in LES samples from patients with achalasia than in those from controls [1]. Additionally, the expression of *ATG16L1* was found to be significantly downregulated at the LES site [1]. In contrast, *IL-1β* expression was significantly upregulated in the LES group [1]. It was hypothesized that HSV1-miR-H1-3p is involved in the induction of *IL-1β* via the downregulation of *ATG16L1*, which was supported by our findings [1]. However, the factors regulating the expression of HSV1-miR-H1-3p could not be identified.

Since CD8^+^ cells suppress the virus, and DOK is known to negatively regulate CD8^+^ cell activation [12,13,14,15], the expression of *DOK* in LES samples was evaluated to confirm the association between DOK and virus reactivation via CD8^+^ cells. The analysis and comprehension of human miRNA expression profiles in the muscular layer of LES are yet to be explored. Therefore, a comprehensive study using microarray analysis and the TargetScan human miRNA database was conducted to validate the association between DOK and human miRNAs.

In this study, the mRNA expression levels of docking proteins 1 and 2 (*DOK1* and *DOK2*) were analyzed and the underlying mechanisms that contribute to the onset of achalasia were investigated.

## 2. Materials and Methods

### 2.1. Ethical Considerations

Written informed consent was obtained from all patients. This study was approved by the Nagasaki University Ethics Committee (approval number: 110328329) and performed following the ethical guidelines of the Declaration of Helsinki.

### 2.2. Peroral Endoscopic Muscular Biopsy Sampling during Peroral Endoscopic Myotomy (POEM)

Standard POEM was performed as previously described [16]. After the submucosal injection, the mucosa was incised, submucosal tunneling and selective myotomy of the internal orbicular muscle were carried out, and finally, the mucosal entrance was closed. General anesthesia was administered and endotracheal intubation was carried out with positive-pressure ventilation in all patients who were subjected to POEM. The study cohort included patients who underwent surgery at Showa University Koto-Toyosu Hospital between October 2011 and June 2012. Patients with severe underlying conditions such as cancer or patients with other conditions for which general anesthesia would be dangerous were excluded. Barium follow-through testing, upper gastrointestinal endoscopy, and pressure measurements were used to diagnose sporadic and classic achalasia. Subsequently, an incision was made in the circular muscle bundle from the entrance to the LES and performed two muscular biopsies using both ends of the biopsy forceps. The biopsy samples were collected from the LES using excised esophagogastric junctions (EGJs) as controls. The control group consisted of patients with esophageal cancer who required surgical resection and whose cancer lesions did not extend to the LES. All control patients were successfully treated with esophagectomy. Immediately after removing the esophagus, including the LES, the resected specimens were longitudinally oriented for further analysis. It is easy to identify the location of the EGJ based on macroscopic findings. Following confirmation by a physician endoscopist with extensive experience in POM, approximately 2 mm of tissue was collected from the internal orbicular muscle using a sharp blade; the LES appeared directly above the EGJ from the mucosal side. High-resolution manometry (HRM) was not used to evaluate the patients in the control group for esophageal peristalsis. However, it was confirmed that medical examinations, including barium follow-through, revealed no symptoms or signs of esophageal dysmotility. Reverse transcription–quantitative (RT-q) PCR was performed on samples from six controls (five males and one female, age range 35–69 years; median age, 66 years) and 11 achalasia patients (seven males and four females; age range 27–78 years; median age, 40 years, including six smokers). Based on *Descriptive Rules for Achalasia of the Esophagus* [17], the achalasia cases were classified into straight-type (8/11) and sigmoid-type achalasia (3/11); one patient was diagnosed with grade I achalasia, and ten patients had grade II achalasia.

### 2.3. RT-qPCR

All samples were immediately placed in 1 mL of RNAlater^®^ reagent (Ambion; Thermo Fisher Scientific, Inc., Waltham, MA, USA) and stored at −80 °C until subsequent RNA isolation. cDNAs were prepared from the total RNA using a high-capacity cDNA Reverse Transcription Kit (Cat. no. 4374966; Thermo Fisher Scientific Inc.). Briefly, reverse transcription reactions were performed using a reaction mixture of 5 μL of total RNA, 1× RT buffer, 4 mM dNTP mix, 1× RT random primers, 50 units MultiScribe™ reverse transcriptase, and 20 units RNase inhibitor, and nuclease-free water was added to a final volume of 20 μL. The reactions were performed at 25 °C for 10 min, 37 °C for 120 min, and 85 °C for 5 min. Primer sequences for quantitative PCR were as follows: *DOK1* forward, 5′-CAATTCTGGGTAACGGTGCAG-3′ and reverse, 5′-CCACCCTCAGCACGTAGGA-3′; *DOK2* forward, 5′-TACGACTGGCCCTACAGGTTT-3′ and reverse, 5′-TCGAACTCAAAGTTGCCCTCT-3′; *ATG16L1* forward, 5′-CAGGCACGAGATAAGTCCCG-3′ and reverse, 5′-AACTCCCCACGTTTCTTGTGT-3′; *IL-1β* forward, 5′-CAGCTACGAATCTCCGACCAC-3′ and reverse, 5′-GGCAGGGAACCAGCATCTTC-3′; and *β-actin* forward, 5′-GCATCCTCACCCTGAAGTA-3′ and reverse, 5′-TGTGGTGCCAGATTTTCTCC-3′. The qPCR reactions were performed in 20 μL aliquots containing 1 μL 10-fold diluted RT product with 4 μL LightCycler^®^ FastStart DNA MasterPLUS SYBR Green I (Cat. No. 03515869001; Roche Diagnostics Co., Ltd., Tokyo, Japan), 0.5 μM of each primer, and 14.6 μL of nuclease-free water. Reactions were performed using a Mic Real-Time PCR Cycler (Cat. no. MIC-2, Bio Molecular Systems Pty., Ltd.). The thermocycling conditions were as follows: denaturation at 95 °C for 10 min, followed by 45 cycles of 95 °C for 10 s, 60 °C for 10 s, and 72 °C for 10 s. The quantification cycle (Cq) was recorded for mRNA amplification using micPCR Software version 2.10.3 (Bio Molecular Systems Pty., Ltd., Tokyo, Japan); *β-actin* was used as an endogenous control for data normalization. Relative expression was calculated using the following formula: 2^−ΔΔCt^ = 2^−(ΔCt, target−ΔCt, endogenous control)^.

### 2.4. miRNA Array Hybridization and Analysis

As previously described [1], total RNAs, including miRNAs from six patients and four controls, were extracted and purified, and each RNA sample was subjected to comprehensive analysis of miR expression patterns using microarray Rel. 16.0 (Agilent Technologies, Santa Clara, CA, USA). Differences in miR expression were considered statistically significant if the fold change in expression values was >2.0 and *p*-value was <0.05.

### 2.5. Statistical Analysis

Differences between two groups were compared using an unpaired two-tailed Student’s *t*-test. Data are presented as box plots, with the minimum, 25th percentile, median, 75th percentile, and maximum values representing the plots. Correlations were calculated using Pearson’s correlation coefficients. The statistical analyses were performed using StatFlex version 7 (Artec Co., Ltd., Osaka, Japan). Statistical significance was set at *p* < 0.05.

## 3. Results

**Figure 1:**
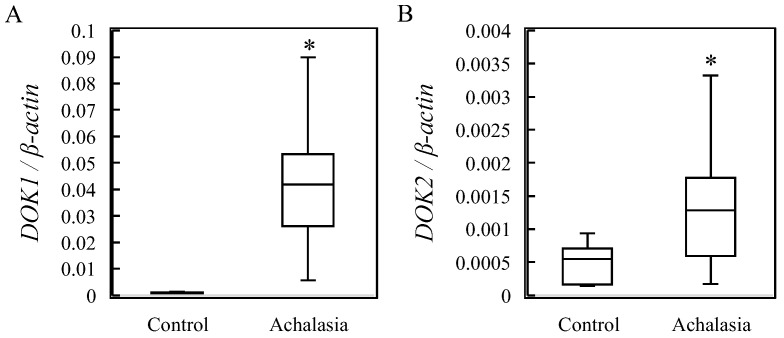
Relative mRNA expression levels in six controls and 11 achalasia patients. mRNA levels of (**A**) *DOK1* and (**B**) *DOK2* using *β-actin* as an endogenous control. * *p* < 0.05. *DOK*, docking protein.

The patient cohort in the current study was the same as that in our previous study [1]. *DOK1* and *DOK2* mRNA levels were significantly upregulated (*p* < 0.05) in the LES of patients with achalasia (Figure 1).

**Figure 2:**
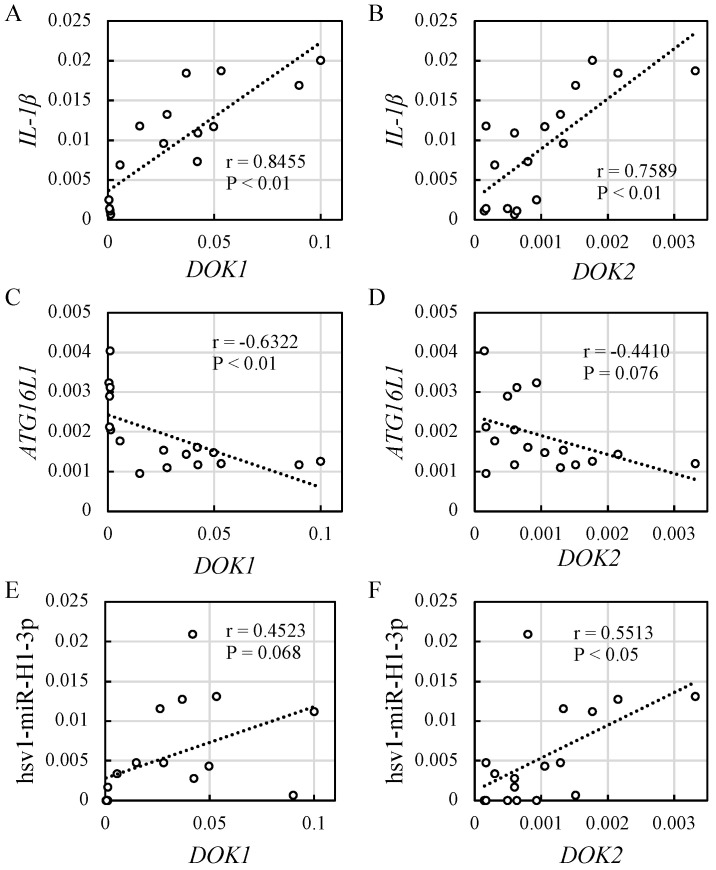
Correlation coefficient analysis in six controls and 11 achalasia patients. Correlation between (**A**,**B**) HSV1-miR-H1 and *DOK* expression, (**C**,**D**) *ATG16L1* and *DOK* expression, and (**E**,**F**) *IL-1β* and *DOK* expression. *DOK*, docking protein; *ATG16L1*, autophagy-related 16-like 1; *IL-1β*, interleukin-1β.

Significant correlations were observed between *IL-1β* and *DOK1* (Figure 2A, r = 0.8455, *p* < 0.01), *IL-1β* and *DOK2* (Figure 2B, r = 0.7589, *p* < 0.01), *ATG16L1* and *DOK1* (Figure 2C, r = −0.6322, *p* < 0.01), and HSV1-miR-H1-3p and *DOK2* expression levels (Figure 2F, r = 0.5513, *p* < 0.05). However, a correlation between *ATG16L1* and *DOK2* (Figure 2D, r = −0.4410, *p* = 0.076) or between HSV-miR-H1-3p and *DOK1* (Figure 2E, r = 0.4523, *p* = 0.068) expression was not found.

**Figure 3:**
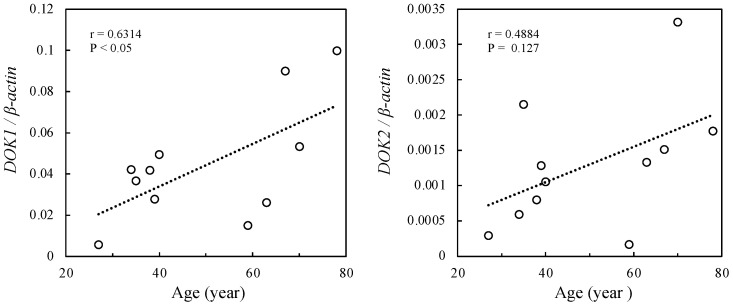
Correlation coefficient analysis. Relationship between *DOK1* and *DOK2* and patient age in 11 achalasia patients. *DOK*, docking protein.

**Figure 4:**
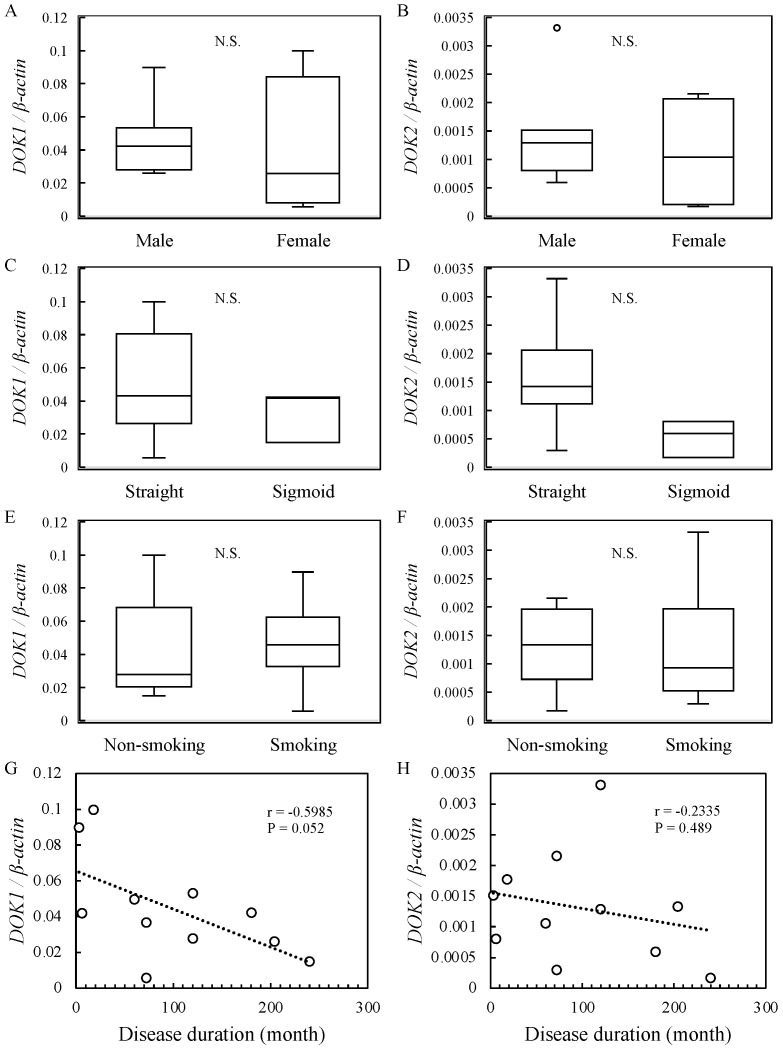
Relative mRNA expression levels and correlation coefficient analysis in 11 achalasia patients. (**A**–**F**) mRNA levels of *DOK1* and *DOK2* using *β-actin* as an endogenous control. (**G**,**H**) Relationship between *DOK1* and *DOK2* and disease duration. *DOK*, docking protein. N.S.: Non Significant.

The relationship between *DOK1* and *DOK2* mRNA expression levels and patients’ clinical parameters was verified. A positive correlation was found between patient age and *DOK1* expression (r = 0.6314, *p* < 0.05) (Figure 3). However, *DOK2* expression levels were not significantly associated with age (Figure 3). There were no significant associations between *DOK1* and *DOK2* mRNA expression levels and sex, achalasia type, smoking status, or disease duration (Figure 4).

**Figure 5:**
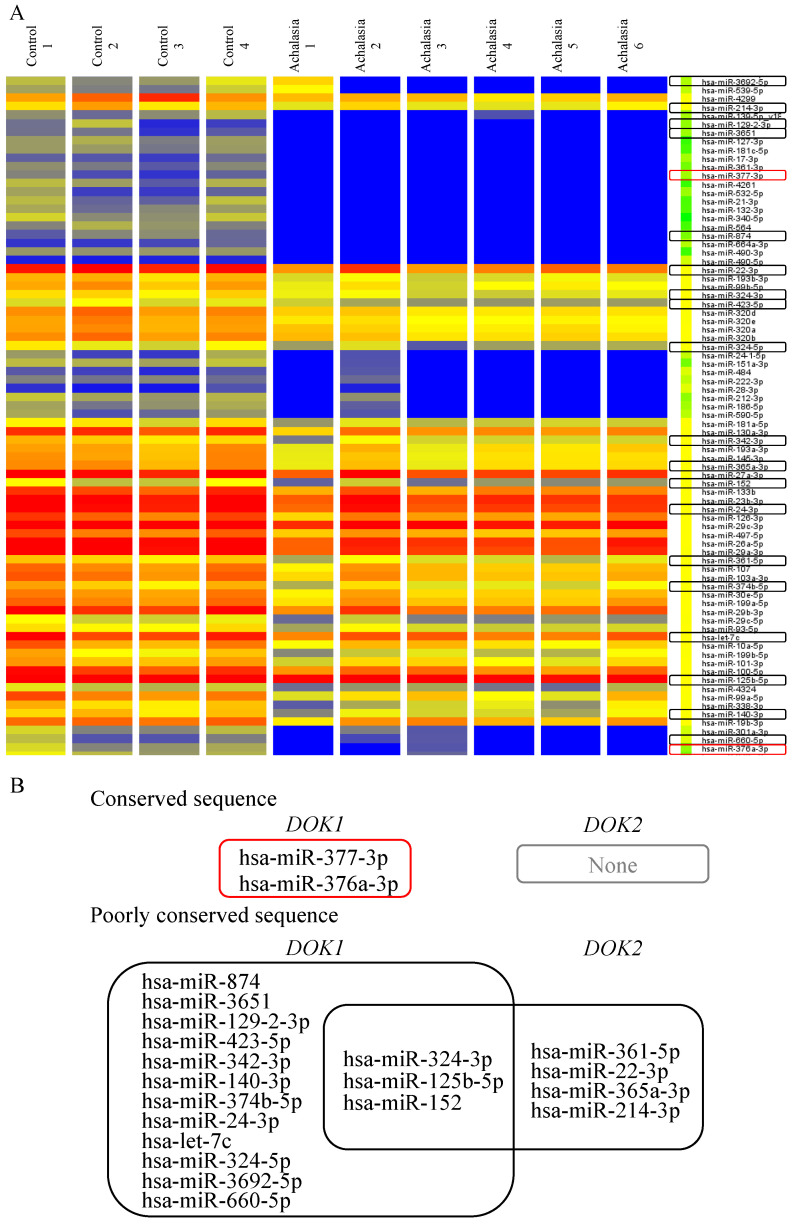
(**A**) Heat map of miRs with downregulated expression in four controls and six achalasia patients. (**B**) The miRs with conserved and poorly conserved sequences targeting *DOK* were extracted from the heat map and shown using red and black enclosures, respectively. *DOK*, docking protein.

In this study, 1205 human and 144 human viral miRNAs were analyzed using a miRNA microarray platform. Using this comprehensive microarray-based technology, the expression profiles of miRNAs in the LES muscle of achalasia patients were characterized and compared with those in the LES of the control group. Microarray analysis revealed differential miRNA expression profiles between the control and achalasia cohorts (Figure 5A). Hsa-miR-377-3p and hsa-miR-376a-3p, highlighted in red in Figure 5, exhibited a significant decrease in expression (*p* < 0.05) in the LES muscle of patients with achalasia. Notably, these miRNAs contain conserved sequences that target *DOK* (Table 1).

## 4. Discussion

Although esophageal achalasia was first reported 300 years ago, its etiology remains unknown. Hence, medical and surgical treatments are aimed at reducing LES pressure. Treatments include endoscopic balloon dilatation, botulinum toxin injection, laparoscopic Heller’s myotomy, and surgical resection of the affected esophagus in advanced cases [4]. POEM has recently been established as a viable and minimally invasive treatment option for esophageal achalasia [16]. This treatment is efficacious and safe, making it suitable for older adult patients, and results in positive short- and long-term prognostic outcomes. Recently, Sato et al. have performed peroral endoscopic biopsies of the muscle layer during POEM, termed POEM-b. According to their study, histopathology and immunohistochemistry of POEM-b samples revealed neurodegenerative signatures rather than inflammatory infiltrates in the muscular layer [18]. Based on HRM and the Chicago classification criteria, type III achalasia exhibits a relatively preserved tendency of interstitial cells in Cajal, whereas type I achalasia shows more severe fibrosis [18,19].

Although the proposed causal factors are diverse and multifactorial, one important factor is HSV-1, as mentioned in the introduction section. We previously reported that HSV1-miR-H1-3p is involved in the induction of IL-1β via the downregulation of ATG16L1 expression [1]. In this study, the mRNA expression levels of *DOK1* and *DOK2* were analyzed to determine the factors regulating the expression of HSV1-miR-H1-3p, because DOK is known to negatively regulate CD8+ cell activation [12,13,14,15].

**Figure 6:**
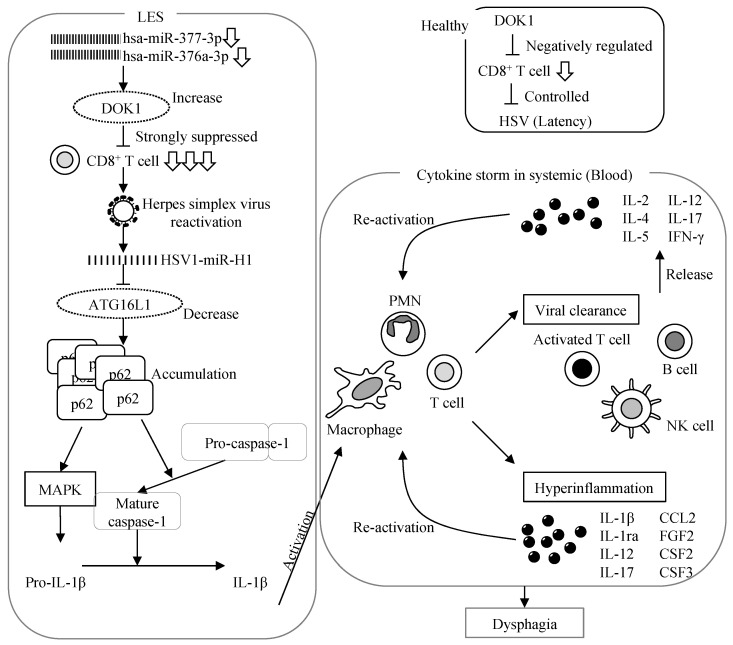
Hypothesis of the underlying mechanism. The decrease in hsa-miR-377-3p and hsa-miR-376a-3p induces DOK expression. DOK suppresses CD8^+^ T-cell activation and induces HSV-1 virus reactivation. HSV-1 releases HSV1-miR-H1. HSV1-miR-H1 decreases *ATG16L1* expression, and the accumulation of p62 is induced by downregulation of *ATG16L1* expression. The abundant p62 induces pro-IL-1β via MAPK activation and activates caspase-1. IL-1β expression is induced by MAPK and caspase. As a result, cytokine storms and dysphagia are induced by IL-1β. DOK, docking protein; ATG16L1, autophagy-related 16-like 1; IL-1β, interleukin-1β.

*DOK1* and *DOK2* mRNA levels were significantly increased in the LES of patients with achalasia (Figure 1). Based on these results, it was hypothesized (see Figure 6) that a reduction in hsa-miR-377-3p and hsa-miR-376a-3p levels leads to the induction of *DOK* expression. In turn, DOK suppresses CD8^+^ T-cell activity [12,13,14,15] and reactivates HSV-1. HSV-1 releases HSV1-miR-H1, which downregulates *ATG16L1* expression [1]. A decrease in ATG16L1 expression results in the accumulation of p62 [20,21]. The abundance of p62 expression, in turn, activates the MAPK pathway and induces the production of pro-IL-1β and activation of caspase-1 [20,21,22,23,24]. Furthermore, the activation of caspase-1 contributes to the conversion of pro-IL-1β to IL-1β [20]. Ultimately, this cascade of events leads to the induction of cytokine storms and dysphagia triggered by IL-1β expression [1] (Figure 6).

Significant correlations were observed between *IL-1β* and *DOK1*, *IL-1β* and *DOK2*, *ATG16L1* and *DOK1*, and HSV1-miR-H1-3p and *DOK2* expressions, supporting our hypothesis (Figure 2 and Figure 6). However, no significant correlation between *ATG16L1* and *DOK2* and HSV-miR-H1-3p and *DOK1* expression was observed (Figure 2). The reason for this observation was presumed to be the timing of the biopsy sample collection, which corresponded to the period when symptoms manifested in patients with achalasia. Therefore, there is the possibility that only the effects of *IL-1β* in the late phase were observed, whereas those of herpes virus miR and *ATG16L* in the early phase were not observed. Moreover, correlations between *ATG16L1* and *DOK2* expression, as well as between HSV-miR-H1-3p and *DOK1* expression were observed. However, no significant associations were found between these factors (Figure 2).

Analysis of the relationship between *DOK* mRNA expression levels and patient clinical parameters revealed a positive correlation between patient age and *DOK1* expression (Figure 3). No significant associations were found between other characteristics and *DOK* mRNA expression levels (Figure 4). Our previous findings [1] confirm the relationship between *IL-1β* expression and age. Therefore, it was predicted that achalasia onset was associated with age. However, *DOK2* expression did not show a significant correlation, which could be due to the timing of biopsy sample collection, as described earlier.

Microarray analysis revealed differential miRNA expression profiles between the control and achalasia cohorts (Figure 5A). The hsa-miR-377-3p and 376a-3p miRs, depicted using red enclosures in Figure 5B, showed significantly decreased expression (*p* < 0.05) in the LES muscle of achalasia patients compared to those in the LES muscles of controls. Additionally, the miR database (TargetScanHuman, https://www.targetscan.org/vert_72/, accessed on 15 May 2024) provided evidence that hsa-miR-377-3p and hsa-miR-376a-3p have conserved sequences that specifically target *DOK*. Furthermore, as represented by the black enclosure in Figure 5B, a few human miRNAs with poorly conserved sequences targeting *DOK* showed significantly decreased expression (*p* < 0.05) in patients with LES compared to those in controls. Moreover, *DOK1* expression was predicted to be more important than *DOK2* expression because the conserved sequences of hsa-miR-377-3p and hsa-miR-376a-3p specifically targeted *DOK1* (Figure 5B). Thus, the expression levels of *DOK1* play a significant role in the manifestation of these symptoms.

Finally, this pilot study had several limitations. Figure 6 illustrates our hypothesis; however, it lacks evidence. First, mRNA expression of *DOK1* was investigated but its protein expression was not. In addition, all samples were used for RNA extraction because of sample size limitations. Therefore, protein analysis could not be performed.

Moreover, in this study, the ability of protein DOK1 to suppress the activity of CD8+ T cells or to reactivate HSV-1 was not demonstrated. Additionally, existing reports explain the series of steps from the accumulation of p62 to pro-IL-1β; however, this aspect was not investigated. To resolve these issues, more samples are needed for protein analysis and other tissue samples, including the trigeminal ganglion for the reactivation of the HSV-1 virus and the muscles of a healthy person, to compare CD8^+^ T-cell activity. The sample size of this study was small, and each sample was obtained from patients diagnosed using classical criteria, primarily based on typical barium findings. A larger sample size and more detailed pathological analysis are needed to overcome these problems in future studies.

Furthermore, in Figure 6, it was hypothesized that some stimulus reactivates the herpes virus in the trigeminal ganglion and releases microRNA into the surrounding area, increasing IL-1β expression in the LES. However, in this study, samples were taken only from the LES; thus, it cannot be proven that DOK expression increased somewhere other than the LES. Tissues from non-motile patients with upper gastrointestinal carcinomas were used as controls, and this did not affect the EGJ, including the LES. This sample was fraught with the following issues: the reduction in hsa-miR-377-3p and hsa-miR-376a-3p levels were described; however, if differences in gene expression induced these reductions, it is difficult to conclude that these miRs are related to achalasia because there are significant gene variations between cancer and non-cancer tissues. Although ideal samples would be obtained from healthy individuals, there are ethical obstacles to obtaining such muscular samples.

Furthermore, achalasia therapy requires an approach from a neural control perspective. As achalasia is a pathological condition characterized by poor muscle movement, POEM from the perspective of muscle contraction was used. However, if muscle movement can be restored to its original state, this could become a fundamental treatment method. This approach is expected to become a necessary treatment method for achalasia in the future but still needs to be verified. One established and validated animal model of achalasia uses adult North American possums. This model establishes an achalasia-like disease by placing a loose Gore-Tex band around the gastroesophageal junction to prevent the relaxation of the lower esophageal sphincter during swallowing. Attempts were made to stimulate the vagus nerve directly and electrically after band removal in this animal model to determine whether it could induce the resumption of peristalsis [25].

Several problems have been mentioned above and one way to overcome them is by establishing animal models. The following model is useful because it shows the pathology of achalasia: it was reported in 1982 that dogs exposed orally to acrylamide develop megaesophagus, thought to be caused by damage to the vagus nerve fibers [26]. Additionally, a transgenic (Pvrl3-Cre) rat strain was developed as a model for achalasia. Ninety percent of transgenic rats developed a megaesophagus at 3–4 months of age. The rats exhibited the classic features of a dilated esophagus, a closed lower esophageal sphincter, and abnormal contractions. Histologically, the lesions in these animals closely resembled those observed in humans. Muscle contractions also demonstrated similarities between the megaesophagus in transgenic rats and patients with achalasia [27]. Another achalasia model consists of local pharmacological denervation of the abdominal portion of the esophagus by wrapping the esophagus of albino rats with thick gauze soaked in the neurotoxin benzalkonium chloride. Apparent morphological and functional disorders were observed in the esophagus of this experimental achalasia model, correlating with changes in observed humans with achalasia [28,29]; another model involves the injection of benzyldimethyltetradecylammonium chloride into the distal esophagus of opossums to increase pressure in the lower esophageal sphincter; some of the histological observations resulting from this treatment resemble features of achalasia [30,31]. Furthermore, a report has shown that aged Rassf1a-deficient mice are more susceptible to megaesophagus than their wild-type littermates. Their gross and histopathological findings closely resembled human cases of megaesophagus/achalasia, indicating that this is a representative mouse model of achalasia disease [32]. However, when using animal models such as those mentioned above, it is essential to note that there are often differences between animals and humans. Triple A syndrome is an autosomal recessive human disorder characterized by adrenal insufficiency, achalasia, alacrimia, and neurological abnormalities affecting the central, peripheral, and autonomic nervous systems. It is caused by mutations in AAAS, which encodes the ALADIN protein. Although a mouse lacking the functional AAAS gene was created, the lack of ALADIN in this mouse did not cause a triple A syndrome-like disease [33].

However, in the pathological model of drug-induced achalasia described above, it is difficult to investigate the focus of our research, i.e., the involvement of herpes viruses. Therefore, a herpesvirus-induced achalasia model is required. Although there are no such animal models, several animal models of herpes sensitization have been developed. An example is the mouse model of latent herpesvirus infection; another example involves a mouse model to investigate HSV-1 reactivation, in which the eyes of mice with corneal injuries are infected with HSV-1, and pooled serum containing HSV-1 neutralizing antibodies is administered intraperitoneally to generate a latent infection mouse model [34,35]. Another experimental system used severely combined immunodeficient mice to determine the T-cell subsets responsible for HSV-1 infection or reactivation, using both corneas in mice inoculated with a virus suspension after making a needle wound [36]. Furthermore, a system in which HSV-1 is applied to each rat nostril has also been established [37].

Herpes virus infection alone cannot induce achalasia; therefore, additional agents are necessary. DOK, hsa-miR-377-3p, and hsa-miR-376a-3p, which we previously reported, are potential targets. In addition, a previous study showed that the truncation mutation of the prion gene PRNP Y162X induced refractory esophageal achalasia [38]. In one case report, a diagnostic workup revealed achalasia in a 7-year-old patient, and whole-exome sequencing revealed a homozygous RBCK1 variant in exon 7. Polyglucosan body myopathy-1 is an extremely rare glycogen storage disease that causes muscle weakness and cardiomyopathy due to the accumulation of polyglucosan bodies [39]. Whole-exome sequencing (WES) on achalasia patients and controls revealed an association between the disease and common missense variants rs1705003 (CUTA) and rs1126511 (HLA-DPB1), and three rare variants (CREB5, ESYT3, and LPIN1) in an independent cohort [40]. A rare sGC variant with a Cys517→Tyr substitution in the α1 subunit of NO-sensitive soluble guanylyl cyclase has been reported to be associated with achalasia [41]. Furthermore, a survey on the susceptibility of mixed-race Mexicans to achalasia revealed that the HLA class II haplotype was a risk factor for achalasia. Thus, an association between achalasia and major histocompatibility complex (MHC) genes has been confirmed [42]. The previously mentioned triple A syndrome includes not only achalasia alone, but a combination of alacrimia and adrenal insufficiency. Therefore, studies on the genetic influence of triple A syndrome may be useful for research on achalasia [43]. This information on genetic mutations will be beneficial for the creation of achalasia models.

Furthermore, in addition to the hypothesis that viral infection causes achalasia, it has also been proposed that an allergenic form of achalasia exists based on the reported infiltration of mast cells in the LES of patients with achalasia [44]. Neural nitric oxide synthase (nNOS) is absent in LES samples obtained from patients with achalasia, and impaired nNOS synthesis in the muscular plexus is considered a significant contributing factor in the development of achalasia [45]. In a rat model with an EGJ incision, the local injection of bone marrow mesenchymal stem cells improved muscle regeneration and increased the contractile function of the injured LES [46]. These findings suggested novel therapeutic targets.

By developing an achalasia animal model using the above models, it would be possible to overcome the limitations of investigating achalasia and its causative factors due to the small number of samples, select more appropriate control samples, and analyze and verify the trigeminal nerve tissues. Furthermore, new treatments can be developed from the perspective of neuromodulation. Creating an achalasia model using a herpes sensitization model is difficult. However, the development of new models is necessary for further analysis and exploration of treatment methods. Furthermore, although analysis and treatment methods using animals cannot necessarily be directly applied to humans, they can significantly advance research.

## 5. Conclusions

The expression of *DOK1* and *DOK2* leads to the induction of *IL-1β* in the LES of achalasia patients, potentially leading to the esophageal motility disorder.

## Figures and Tables

**Table 1:** Conserved sequence of *DOK1*, and miRs are underlined. Sequence data were obtained from the TargetScan Human miR database.

	Predicted Consequential Pairing of Target Region (Top) and miRNA (Bottom)
Position 1172-1179 of *DOK1* 3′ UTR	5′ …UUUAAGAAGUUUAUG-UGUGUGAA...
hsa-miR-377-3p	3′ UGUUUUCAACGGAAACACACUA
Position 363-369 of *DOK1* 3′ UTR	5′ …CCAAAGAGGAUCCCA-UCUAUGAU...
hsa-miR-376a-3p	3′ UGCACCUAAAAGGAGAUACUA

## Data Availability

All data generated or analyzed during this study are included in this article, and we used information from TargetScanHuman 7.2 (https://www.targetscan.org/vert_72/, accessed on 15 May 2024).
